# Targeting pan-essential pathways in cancer with cytotoxic chemotherapy: challenges and opportunities

**DOI:** 10.1007/s00280-023-04562-3

**Published:** 2023-07-15

**Authors:** Sean G. Rudd

**Affiliations:** Science For Life Laboratory, Department of Oncology-Pathology, Karolinska Institutet, Stockholm, Sweden

**Keywords:** Cytotoxic chemotherapy, Conventional chemotherapy, Therapeutic index, Precision medicine, Molecular mechanism of action

## Abstract

Cytotoxic chemotherapy remains a key modality in cancer treatment. These therapies, successfully used for decades, continue to transform the lives of cancer patients daily. With the high attrition rate of current oncology drug development, combined with the knowledge that most new therapies do not displace standard-of-care treatments and that many healthcare systems cannot afford these new therapies; cytotoxic chemotherapies will remain an important component of cancer therapy for many years to come. The clinical value of these therapies is often under-appreciated within the pre-clinical cancer research community, where this diverse class of agents are often grouped together as non-specific cellular poisons killing tumor cells based solely upon proliferation rate; however, this is inaccurate. This review article seeks to reaffirm the importance of focusing research efforts upon improving our basic understanding of how these drugs work, discussing their ability to target pan-essential pathways in cancer cells, the relationship of this to the chemotherapeutic window, and highlighting basic science approaches that can be employed towards refining their use.

## Introduction

Cytotoxic chemotherapy, also referred to as conventional or classical chemotherapy, is a central pillar of cancer treatment and has been for many decades [1, 2]. These therapies, particularly when used in combination, have high clinical impact and continue to transform the lives of cancer patients on a daily basis. This can sometimes be unappreciated within the pre-clinical cancer research community, in which we often present these therapies as a group of non-specific cellular poisons, especially when contrasted with new molecularly targeted therapies in this era of precision oncology. There are of course spectacular success stories [3, 4] that motivates an ever expanding list of drugs to enter clinical studies, but we have a disproportionately high failure rate of these new therapies [5]. Whilst the reasons for this are many and increasing efforts are focused to understand this [6–9], it is important to note that most agents that do successfully progress through clinical testing do not actually displace standard-of-care therapies. Instead, they are used in combination with standard-of-care, or as an option for those patients who have exhausted previous treatment options [10]. We should also not neglect that new agents are expensive [11] and many healthcare systems and patients will not have access to these and will rely on the more affordable alternative of conventional chemotherapeutic agents. A recent survey questioned frontline oncologists across the globe for a list of the top 20 cancer medicines deemed essential to their practice, and of these, 12 were cytotoxic agents [12].

This is not written as a criticism of developing new molecules or therapeutic modalities, as this is vital, but rather as an argument to reassess the importance of cytotoxic chemotherapies, and in particular, the importance of focusing research efforts upon improving our basic understanding of how these drugs work. Knowledge gained could provide the scientific basis for refining the use of these therapies, which could have immediate clinical impact worldwide. Furthermore, an argument could be made that one factor contributing to the disproportionately high failure rate of new targeted therapies is a misunderstanding of how current standard-of-care therapies, principally cytotoxic chemotherapies, work as medicines. Understanding the mechanistic principles underlying successful treatments, even those which are half a century old, is critical for improving new therapies. Thus, in this article, I wish to reaffirm the importance of improving our basic understanding of these therapies together with highlighting several unanswered questions along with current approaches that can be employed towards refining their use.

## A diverse group of agents with distinct molecular mechanisms

Cytotoxic chemotherapy is a broad term which encompasses an array of anticancer drugs with activities in many distinct malignancies. Often, this term is used to associate therapies with non-specific mechanisms of action, so-called “dirty drugs”, considered to be generally cytotoxic to highly proliferative tissues and this being the main basis of obtaining a therapeutic window. We now know that this is a vast oversimplification, as will be discussed below; however, this (mis)information is constantly perpetuated in our research literature and in the information given to cancer patients.

What these agents have in common is that they were identified somewhat empirically (although phenotypic rationales for cancer selectivity existed [1, 2]), using then state-of-the-art approaches, and were shown to have effects in pre-clinical cancer models before progressing (often rather quickly) into patients; however, their molecular mechanism of action was not defined at that time. This is often discussed in contrast to the traditional view of targeted therapies that can start with a (hopefully) validated target in mind, which is far from trivial [13, 14], and molecules are subsequently identified to modulate this target in cancer cells; thus ideally, the target and anti-cancer mechanism is defined from the outset (thus, informing mechanism-based use of the therapy). This is despite a substantial amount of new therapies resulting from phenotypic drug discovery [6, 15]. Looking from our current standpoint, many decades after the identification and subsequent introduction of many chemotherapeutics into the clinic, we have vastly increased our understanding of their pharmacology together with the fundamentals of cancer biology. We now know that many of these agents have distinct molecular mechanisms of action, targeting known cancer cell dependencies or perturbing essential biochemical pathways, which is also why these agents have more recently been referred to as *targeted* cytotoxic therapy [16].

Antimetabolites, a group of chemotherapies that were amongst the first to show clinical success in treating cancer [17], are an excellent case study for highlighting the distinct molecular mechanisms of action of chemotherapeutics in targeting well-established cancer cell dependencies (even within a single drug class) [18]. Nucleotide biosynthesis can be regarded as a non-oncogene addiction of cancer cells, as DNA building blocks (deoxynucleoside triphosphates, dNTPs) are required to fuel elevated genome duplication and repair. Antimetabolites, which are synthetic mimics of nucleosides or folate, can be potent and specific inhibitors of enzymes within these pathways and thus starve cancer cells of specific substrates for DNA synthesis [18]. Additionally, many of these compounds can directly perturb DNA metabolism through distinct molecular mechanisms, for instance by slowing/stalling the DNA synthetic reaction, inducing lesions to trap cancer cells in futile DNA repair cycles, or by causing DNA–protein crosslinks [18]. Thus, this family of compounds—via distinct mechanisms—effectively exploit two valid anticancer targets, DNA precursor metabolism and genomic integrity [19]. Similar examples can be taken from other classes of cytotoxic chemotherapy, with genomic integrity being a common target [20]. Alkylating agents can induce a spectrum of base modifications which are metabolized and repaired in distinct ways [21], whilst platinum-based agents crosslink DNA strands to inhibit normal DNA function [22] [23], both allowing exploitation for tumor cell killing. Topoisomerase poisons trap the enzymes responsible for releasing torsional strain during DNA metabolism on the DNA molecule during catalysis, inducing strand breaks coupled with a DNA–protein crosslink, a potent cytotoxic lesion [24]. Specifically, topoisomerase I is the cellular target of camptothecins, which act at the level of the DNA–topoisomerase I complex and, through stabilization of this complex, stimulate DNA cleavage. Topoisomerase II poisons (e.g., anthracyclines) act by inhibiting the religation of DNA–topoisomerase II complexes, whereas others induce their formation [25]. To summarize, these agents induce distinct DNA lesions that are metabolized by cancer cells in specific ways (via replication, transcription, and/or repair), dependent upon cellular context, with specific cellular outcomes. However, despite the detailed molecular mechanisms of action we have mapped out over several decades of research, there is much left to be understood which can impact upon the clinical use of these therapies, from further refinement of those mechanisms to, more broadly, how these therapies work as medicines.

## “The proliferation rate paradox”

The name for this section is taken from a thought-provoking perspective article [26] which highlights a largely unanswered question in the cancer research community: how do cytotoxic chemotherapies work as medicines? As overviewed in the previous section, we now have a detailed understanding for the molecular underpinnings of the mechanism of action for many of these therapies from work in pre-clinical cancer models (which continues to be refined through state-of-the art experimental approaches, exemplified in refs [27, 28]), but what is the relationship of this understanding to their mechanism of action as medicines?

The “proliferation rate paradox” questions the widely perpetuated view that cytotoxic chemotherapies are effective anti-cancer medicines owing to their ability to target highly proliferative tissues, which cancers are typically thought to be, thus offering a therapeutic window owing to slower growing non-malignant tissues. Non-malignant tissues often responsible for dose-limiting toxicities of cancer drugs can be the bone marrow and gut crypts, those tissues with the highest cellular turnover [29], with doubling times for the bone marrow, for instance, reported to range from 17 h to 3 days. However, analysis of tumor doubling rates, which varies widely dependent upon tumor type (and can vary at different locations within the same tumor), can range from one week to over a year [30]. This discrepancy is by no means a new discovery, as pointed out by Mitchison [26], studies in the 1970s began asking the same question and assembled data to provide answers [31, 32]. And over the subsequent years, several articles have highlighted this problem again and compiled evidence from various sources [26, 33, 34]. Tumor doubling rates will be a measure of both cell proliferation and cell death, but it is clear that tumors can be very slow growing, and analysis of the proportion of S-phase cells in tumors also supports this [26], as does the striking array of cell-cycle phases observed in a recent analysis of multiple human cancers [35]. This is potentially further supported by the limited clinical utility of nucleotide-derived positron emission tomography (PET) tracers to visualize tumors (as these are dependent upon replicating cells) [36], as highlighted by Yan and colleagues [34]. In addition, a recent analysis of publicly available data from the Human Protein Atlas of Ki67 staining (a proliferation marker) in normal and malignant tissue also underscored that dose-limiting non-malignant tissues can have a higher proliferative index than malignant counterparts [34]. This is one of the suggested reasons for the failure of some cell-cycle-targeted therapies (such as mitotic kinase inhibitors) in clinical studies, as if drugs kill solely based upon proliferation rate, on-target dose-limiting toxicity will prevent efficacy [8, 26, 33, 34].

## The therapeutic window

**Fig. 1:**
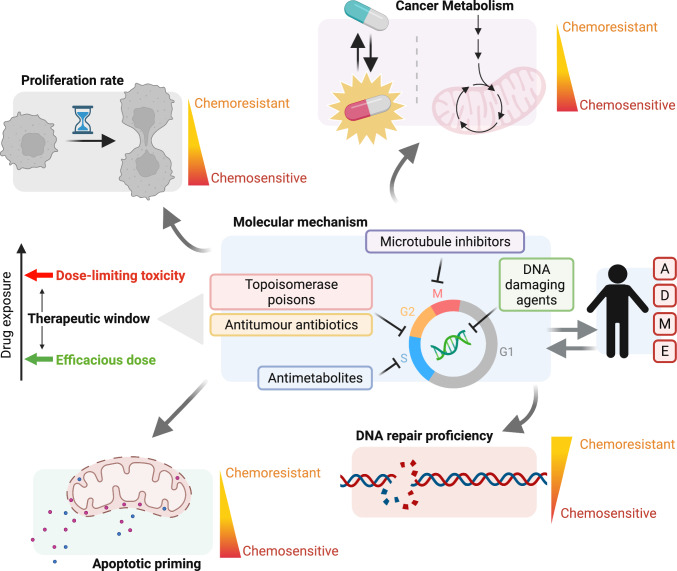
The chemotherapeutic window. Schematic representation of factors reported to determine the therapeutic window of cytotoxic chemotherapies discussed in this manuscript. Figure created with Biorender

Considering the above information, if cytotoxic chemotherapies do kill cells based solely upon proliferation rates, how would they achieve selectivity for malignant versus non-malignant tissue (especially if cell-cycle kinase inhibitors cannot)? Rather than solely proliferation rate, there is likely several reasons why cytotoxic chemotherapies can achieve therapeutic windows, overviewed in Fig. 1, and perhaps (to some extent) these reasons will be specific to individual classes of cytotoxic chemotherapy owing to their distinct molecular mechanisms of action.

DNA repair and cell-cycle checkpoint proficiency have been suggested to account for the therapeutic window of some cytotoxic chemotherapies. A common target for many of these drugs is the DNA molecule with different drugs inducing different DNA lesions [20] and thereby cells rely on distinct repair pathways to remove the resulting DNA damage in an attempt to restore genomic integrity. Cancer cells can often be defective in repair pathways, which although fuels cancer development through acquisition of mutations and genomic rearrangements [37], also renders these cells differentially sensitive to DNA damaging agents compared to their non-malignant counterparts. Within pre-clinical research there are a plethora of examples [20], including thiopurine cytotoxicity being dependent upon proficient mis-match repair [38], inactivation of PrimPol-mediated DNA damage tolerance sensitizing to inter-strand crosslinking agents [39], and dependency upon proficient homologous recombination (HR) repair to fix cytotoxic DNA double-strand breaks caused by replication of chemotherapy-induced DNA lesions [40]. Clinical evidence is particularly strong for the effectiveness of platinum agents in HR defective (e.g., BRCA-mutant) cancers [41–43]. Cancer cells also have higher levels of replication stress [44, 45] and thus agents which exacerbate this, which are many amongst cytotoxic chemotherapy, could selectively kill cancer cells over non-malignant cells [46].

With regards to antimetabolites, differences between normal and cancer cell metabolism have been suggested to be responsible for the clinical success of these therapies [47]. It is well established that cancer cells rewire their metabolism to support biomass production, which can be dependent upon several factors, and it is possible these changes render cells selectively sensitive to antimetabolite therapies. This is consistent with the initial phenotypic observations that spurred the development of some of these drugs (e.g., cancer cells increase uptake and use of uracil from medium being the basis of 5-fluorouracil development [2]) and more recent examples also exist. For instance, a recent study highlighted that a subset of lung cancers are dependent upon pyrimidine salvage pathways, which explained the lack of pre-clinical models being sensitive to inhibition of de novo pyrimidine synthesis via inactivation of the enzyme DHODH [48] (as these are the complimentary pathways that can supply pyrimidine nucleotides, de novo and salvage). Pyrimidine analogs fluorouracil and gemcitabine can be effective in these cancers, and these therapies are prodrugs, requiring activation by these same pyrimidine salvage pathways.

An extension of this argument was recently made by Yan and colleagues [34] which pointed out that many of the cytotoxic chemotherapies used in the clinic are actually prodrugs. In addition to antimetabolites, this includes methylating agents, nitrogen mustards, and platinum-based agents (for thorough drug list see ref [34]), and these compounds thus undergo intracellular activation to exert their antitumour properties, and, as highlighted by the authors, many of the pathways or conditions needed to bioactivate these compounds can be elevated in certain cancer types, which could potentially account for a therapeutic window. However, other cytotoxic chemotherapies, such as the microtubule targeting agent paclitaxel, are not prodrugs but can also successfully treat solid malignancies before encountering dose-limiting toxicities, which intriguingly, targeted mitotic kinase inhibitors cannot [8, 33, 34]. The discrepancy between the utility of paclitaxel versus targeted mitotic kinase inhibitors can potentially be explained by the molecular mechanism of action of paclitaxel in tumors, where concentrations are much lower than what is typically utilized in cell culture experiments, and rather than inducing mitotic arrest (which is the desired phenotype for mitotic kinase inhibitors), it is chromosome mis-segregation on multipolar spindles (without mitotic arrest) that is the clinically relevant phenotype [49]. However, why paclitaxel can target malignant tissue over its non-malignant counterparts still appears to be an unanswered question [50].

Whilst all reasons discussed thus far have been tailored to the distinct molecular mechanisms of these therapies, Letai and colleagues provided a broader explanation that can encompass all (cytotoxic) chemotherapy, termed mitochondrial or apoptotic priming, first reported over a decade ago [51, 52]. Apoptotic priming is based upon the principle that most chemotherapies kill cells via the intrinsic mitochondrial-dependent pathway of apoptosis, and different tissues (both malignant and non-malignant) have a different propensity to execute this pathway owing to how close the mitochondria within those tissues are to this apoptotic threshold (i.e., mitochondrial outermembrane permeabilization, MOMP). Accordingly, it was shown that the mitochondria within chemosensitive tissues, whether malignant or non-malignant, where closer to this apoptotic threshold (i.e., primed), whilst mitochondria in tissues known to be typically chemoresistant where further from this threshold (i.e., not primed), explaining differential sensitivity and thus the therapeutic window. This phenomenon also explained why some tumors are generally chemosensitive regardless of the therapy used, childhood acute lymphoblastic leukemia being a prime example, as these malignant cells are closer to the apoptotic threshold, whilst others are generally chemoresistant.

## Targeting pan-essential genes vs pathways

A pan-essential gene can be defined as a gene which, if lost, results in a loss of cell fitness or death in multiple normal tissues or cell lineages [8], which can now be readily identified through publicly available genome-wide CRISPR knockout screens [53]. Whilst several cytotoxic chemotherapies can be potent inhibitors of known pan-essential genes, such as methotrexate targeting dihydrofolate reductase (DHFR) or gemcitabine covalently inhibiting ribonucleotide reductase (RNR), these therapies can often be polypharmacologic, and many can target more broadly a metabolic process (such as perturbing the DNA synthetic reaction in a particular way). Thus, it is perhaps more accurate to consider these therapies as ones which target pan-essential pathways. With this in mind, a recent perspective article [8] discussed in depth the pitfalls of developing cancer drugs targeting pan-essential genes (such as the cell-cycle inhibitors discussed above), and many lessons learnt were presented that should be applied to the development of future therapies. Given the similarity of targeting pan-essential genes to cytotoxic chemotherapies targeting pan-essential pathways, which was noted by the authors [8], one point that was not discussed was that much of the knowledge and approaches outlined by the authors could also be applied to conventional cytotoxic chemotherapies.

For instance, several key features of successful targeted therapies (i.e., those with a high therapeutic index) were listed [8], and a number of these features can be found in pre-clinical or clinical data regarding cytotoxic chemotherapies, highlighting potential avenues to refine their use. Lineage-restricted therapies are those which target a particular cell lineage regardless of whether it is malignant, a prime example being B-cell targeted therapies such as Brunton’s Tyrosine Kinase (BTK) inhibitors, which successfully treat B-cell malignancies whilst also killing normal B cells [54]. The antimetabolite nelarabine, a guanosine analog, was developed following the observation that build-up of the metabolite deoxyguanosine triphosphate (dGTP) was selectively toxic to T cells. This selectively was shown to be the case for nelarabine too and now this therapy is approved for use in relapsed and refractory T-cell malignancies [55].

Synthetic lethality was another key feature of high-therapeutic index therapies, the concept in which loss of two complimentary pathways is required for cell killing [56], the quintessential example being the use of PARP inhibitors in BRCA-mutated cancers [57, 58]. This feature has also been reported with several chemotherapeutics, although best described as hypersensitivity. BRCA-mutated cancer models are also hypersensitive to the antimetabolite 6-thioguanine owing to this compound inducing cytotoxic DNA damage during DNA synthesis requiring HR repair [59]. Furthermore, 6-thioguanine could overcome platinum and PARP inhibitor resistance in pre-clinical models [59]. This was also shown to be the case for alkylating agents [60, 61]. With regards to “*BRAF*-like” colon cancer, genome-wide shRNA screening revealed a selective vulnerability during mitotic progression when compared to non-*BRAF*-like colon cancer, which can be successfully targeted with the microtubule poison vinorelbine [62]. Another example comes from analysis of exceptional responders that identified that tumors with a defective DNA damage response display synthetic lethality with temozolomide [63].

Use of predictive biomarkers, which allow focused use of a treatment in those patients with a high probability of response, is another feature of high-therapeutic index therapies [8, 64]. In addition to the synthetic lethal examples outlined above, harnessing knowledge on chemotherapy metabolism can also be advantageous. Prodrugs like antimetabolites require activation by a cascade of enzymes to elicit their anticancer effect, and these drugs are also subject to catabolic processes, all of which impacts the efficacy/toxicity balance, which has been discussed in detail [18, 65]. Recent examples include the nucleotide hydrolyses SAMHD1 and NUDT15 that can convert the active metabolites of several nucleoside analogs into inactive forms. In the case of SAMHD1 and the deoxycytidine analog cytarabine, which is standard-of-care in acute myeloid leukemia, this has implications on treatment efficacy [66–68], whilst in the case of NUDT15, enzyme variants are associated with increased toxicity following thiopurine treatment [69, 70], offering the basis for dose individualization [71].

Another key feature of high-therapeutic index therapies are those which can exploit differential surface-antigen expression [8], and whilst cytotoxic chemotherapy alone is unable to do this, antibody–drug conjugates that utilize cytotoxic payloads do offer this advantage. For example, derivatives of the topoisomerase poison camptothecin and microtubule inhibitors have been used as payloads on multiple antibody–drug conjugates [72], offering the potential to target these chemotherapeutics to specific cellular subsets. Despite many open questions within this field—offering potential for optimization—there is evidence that this therapeutic modality can offer improved efficacy over cytotoxic chemotherapy [73]. There are also efforts to broadly target cytotoxic chemotherapies to cancer cells in an antigen-independent manner, for instance by exploiting consequences of the Warburg effect [74].

## Refinement of cytotoxic chemotherapy: a focus on molecular mechanism

Several strategies have been employed to optimize chemotherapy treatments, to increase their therapeutic window, reviewed by Chang et al. [8]. These include schedule optimization (for instance on–off dosing strategies), the use of supportive medicine to mitigate treatment side effects, optimization of chemotherapy formulation to shift drug distribution towards the tumor, innovative drug combinations, and personalizing dosing based upon body surface area, weight, and renal function. These approaches, in some cases with several decades of documented use, have been successful in increasing the chemotherapeutic window. However, there is much room to exploit the molecular mechanisms of these therapies further, and to match this with the molecular characteristics of the patient and their cancer, akin to efforts for therapies targeting pan-essential genes [8]. Thus, it is tempting to speculate additional advances can be made and one key component of this will be to improve our basic understanding of how these therapies work. This information provides the basis of hypotheses that can be retrospectively evaluated using clinical data, if available, given the widespread use of these agents, or alternatively new clinical studies could be established.

For instance, exploiting large-scale pharmacogenomic datasets, whether pan-cancer [75, 76] or disease-focused [77], in which panels of cancer cell lines thoroughly characterised with various omic technologies (transcriptome, proteome, metabolome, etc.) are subject to an array of drug perturbations, can be a powerful approach to identify correlates of drug efficacy. Expansion of these datasets to encompass more advanced near-patient models, such as those employed in functional precision medicine efforts [78], is a particularly exciting prospect, especially when combining with controls relevant to understanding the therapeutic window (i.e., those tissues which are associated with dose-limiting toxicities). Coupling pharmacogenomic datasets with data derived from genome-wide CRISPR loss-of-function screens can also allow deciphering of drug mode of action [79], although the polypharmacologic nature of some cytotoxic chemotherapies may complicate this. Altogether, these studies facilitate identification of putative predictive and pharmacodynamic/pharmacokinetic (PD/PK) biomarkers, which can be interrogated in subsequent focused studies. Given many of these therapies are prodrugs [34] and subject to large inter-individual variability in PK, identification of PD/PK biomarkers could facilitate dosing and schedule optimisation, which can be an important aspect of refining treatments. Furthermore, these efforts could allow identification of potential therapeutic targets to enhance treatment efficacy, for instance by targeting those factors associated with treatment resistance [80].

Findings from such studies will undoubtably require validation, and here it is key that methods and models appropriate to the therapy and malignancy in question are used together with employing more complex modes of drug testing. For instance, frequently, measurement of ATP in cell lysate is used as a proxy for quantifying viable cells following drug exposure, however if drug treatment alters cell size or affects ATP metabolism, data from these readouts will be misleading [81]. Additionally, drug response is typically characterised following 3-day continuous exposure, however more complex dose-scheduling may be warranted, especially with a focus upon pharmacologically relevant drug doses [82]. Employing these approaches with a thorough assessment of drug efficacy readouts—not just IC_50_ values—can also yield valuable information [83], and it is important to account for possible confounding factors such as differing proliferation rate [84]. Coupling such approaches with single-cell multi-parameter readouts, as recently exemplified [85], can be particular powerful in characterizing drug responses in single cells yielding information-rich datasets. These focused approaches can yield unexpected and clinically relevant biology of cytotoxic chemotherapies. For instance, analysis of long-term single-cell responses to cisplatin-exposed cells found an unexpected relationship between proliferation rate and cell killing, with highly proliferative cells being more likely to arrest than die, whilst the opposite was observed for slowly proliferating cells [86]. Similarly, coupling super-resolution microscopy with a clickable analog of the antimetabolite cytarabine, also revealed an unexpected relationship between replication and drug toxicity, finding drug-resistant cells can incorporate more of this analog into genomic DNA whilst sensitive cells incorporated less [87].

Given the complexity of (cancer) biology and its interaction with small molecules, especially those which require metabolism (which is many amongst cytotoxic chemotherapies [34]), hypothesis-free unbiased approaches will be key in furthering our molecular understanding of these clinically used therapies. In addition to harnessing large-scale pharmacogenomic datasets, discussed above, this includes approaches such as pooled whole-genome CRISPR screens to identify therapy resistance and sensitization factors [88]. These can interrogate many cytotoxic agents within the same cell models, identifying both common and drug-specific pathways [89, 90], although as models used will not always be relevant to all drugs evaluated, findings specific to the biology of cancer subsets will be omitted. Alternatively, cell models representing specific malignancies can be used and drugs relevant for this cancer screened [91, 92], yielding more disease relevant datasets. Identification of drug-resistant alleles is often considered the gold-standard of identifying a drugs target [93], and although when considering the polypharmacology associated with some cytotoxic agents which could complicate data interpretation, methods developed to identify drug-resistant alleles have been utilised successfully with cytotoxic agents under pre-clinical and clinical investigation [94, 95]. Whilst these approaches have not been extensively used on current cytotoxic chemotherapies used in the clinic, examples exist, such as the use with thioguanine which successfully identified a previously known key metabolic enzyme [96]. This highlights that with this family of compounds, key drug-resistant alleles could be those catalyzing an early metabolic step required for drug activation, in line with identification of deoxycytidine kinase in CRISPR screening efforts against multiple nucleoside analogs [89].

Chemoproteomic approaches are another powerful approach to gain insight into the molecular mechanisms of drugs [97]. Thermal proteome profiling, exploiting the simplicity of a thermal shift assay but on a proteome scale, allows unbiased mapping of protein stability and abundance changes within the proteome of drug-exposed cells [98, 99], which was recently exemplified with cytotoxic agent 5-fluorouracil uncovering new biology associated with this decades old therapy [27]. Another key unbiased approach to defining the molecular mechanism of therapeutics is morphological cell profiling, or cell painting [100], when applied to drug-exposed cells. The power of this technology in deciphering drug mechanism is particularly strong when it is applied to libraries of compounds [101, 102]; however, as discussed previously for CRISPR screening, this can prevent the use of disease-specific models and may hinder identification of disease-specific biology relevant to the drug molecular mechanism. This can of course be overcome by focusing research efforts on models representing malignancies of interest, if this is the purpose of the study.

Knowledge gained with the approaches discussed above will inform the rational design of drug combinations—which is key [103]—ideally therapies with monotherapy efficacy and a high-therapeutic index. Combing therapies serves multiple purposes, it can be used to enhance cancer cell killing, reduce treatment toxicity, and/or prevent the onset of treatment resistance; combinations of agents is how cancer is successfully treated. Although much focus in pre-clinical research centers upon finding synergistic combinations, there are data clearly arguing that this is not necessary for clinical benefit [104, 105], and instead research efforts should focus on combining independently active drugs with resistance mechanisms that do not overlap, to maximize anti-cancer efficacy within the context of tumor heterogeneity [106]. Combinations with immune-oncology therapies is also an important avenue being explored [107]. Although cytotoxic chemotherapy has long-been considered to be immune suppressive, there are increasing data supporting the efficacy of these therapies involves activation of antitumour immune responses [108].

## Conclusions and future perspectives

Improving our understanding of clinically active therapeutics is key to rationally refining their use and improving patient responses, whether in a patient population or in efforts to individualize treatments. When considering the high attrition rate in current oncology drug development coupled with the knowledge that most new therapies do not displace standard-of-care treatments, and the high financial burden of new therapies often preventing worldwide use, it is clear that cytotoxic chemotherapies are going to remain an important component of cancer therapy for many years to come. It is, thus, important to focus research efforts upon these tried-and-tested therapies. As outlined here, these are not a group of non-specific cellular poisons killing cells based solely upon proliferation rate, but a diverse group of anticancer agents with distinct molecular mechanisms that target pan-essential pathways in cancer cells. The more we learn about these therapeutics, the unappreciated intricacies of their modes of action, the line between cytotoxic chemotherapies and subsequently developed targeted agents becomes increasingly blurred, revealing a broad spectrum of clinically active agents, which should all be taken full advantage of. By furthering our knowledge on the molecular mechanisms underpinning the activity of these compounds, and the relationship of this to the factors that dictate the chemotherapeutic window (Fig. 1), we can continue towards the refinement and optimisation of the clinical use of these therapies. Furthermore, understanding the mechanistic principles of current therapies could also provide a strong foundation for the development of new effective therapies. For example, with the knowledge that the clinical success of paclitaxel is owing to dysregulation of mitosis but without actual mitotic arrest [49], new antimitotic agents could be developed with this goal in mind, perhaps overcoming previous clinical failures in this area [8, 34]. Whilst another example could be embracing the prodrug strategy that is abundant in clinically successful conventional agents but underexplored with new therapeutics [34].

## Data Availability

Data sharing not applicable to this article as no datasets were generated or analyzed during the current study.
